# Regional trends in birth weight in low- and middle-income countries 2013–2018

**DOI:** 10.1186/s12978-020-01026-2

**Published:** 2020-12-17

**Authors:** Irene Marete, Osayame Ekhaguere, Carla M. Bann, Sherri L. Bucher, Paul Nyongesa, Archana B. Patel, Patricia L. Hibberd, Sarah Saleem, Robert L. Goldenberg, Shivaprasad S. Goudar, Richard J. Derman, Ana L. Garces, Nancy F. Krebs, Elwyn Chomba, Waldemar A. Carlo, Adrien Lokangaka, Melissa Bauserman, Marion Koso-Thomas, Janet L. Moore, Elizabeth M. McClure, Fabian Esamai

**Affiliations:** 1 Moi University School of Medicine, Eldoret, Kenya; 2 Division of Neonatal-Perinatal Medicine, Indiana University School of Medicine, Indianapolis, IN USA; 3 RTI International, Durham, NC USA; 4 Lata Medical Research Foundation, Nagpur, India; 5 Boston University School of Public Health, Boston, MA USA; 6 Aga Khan University, Karachi, Pakistan; 7 Department of Obstetrics and Gynecology, Columbia University School of Medicine, New York, NY USA; 8 KLE Academy Higher Education and Research, J N Medical College Belagavi, Karnataka, India; 9 Thomas Jefferson University, Philadelphia, USA; 10 Instituto de Nutrición de Centroamérica y Panamá, Guatemala City, Guatemala; 11 University of Colorado School of Medicine, Denver, CO USA; 12 University of Alabama at Birmingham, Birmingham, AL USA; 13 University Teaching Hospital, Lusaka, Zambia; 14 Kinshasa School of Public Health, Kinshasa, Democratic Republic of Congo; 15 University of North Carolina at Chapel Hill, Chapel Hill, NC USA; 16 Eunice Kennedy Shriver National Institute of Child Health and Human Development, Bethesda, MD USA

**Keywords:** Birth weight, Global network, Low birth weight, Neonatal mortality, Newborns

## Abstract

**Background:**

Birth weight (BW) is a strong predictor of neonatal outcomes. The purpose of this study was to compare BWs between global regions (south Asia, sub-Saharan Africa, Central America) prospectively and to determine if trends exist in BW over time using the population-based maternal and newborn registry (MNHR) of the Global Network for Women'sand Children's Health Research (Global Network).

**Methods:**

The MNHR is a prospective observational population-based registryof six research sites participating in the Global Network (2013–2018), within five low- and middle-income countries (Kenya, Zambia, India, Pakistan, and Guatemala) in threeglobal regions (sub-Saharan Af rica, south Asia, Central America). The birth weights were obtained for all infants born during the study period. This was done either by abstracting from the infants' health facility records or from direct measurement by the registry staff for infants born at home. After controlling for demographic characteristics, mixed-effect regression models were utilized to examine regional differences in birth weights over time.

**Results:**

The overall BW meanswere higher for the African sites (Zambia and Kenya), 3186 g (SD 463 g) in 2013 and 3149 g (SD 449 g) in 2018, ascompared to Asian sites (Belagavi and Nagpur, India and Pakistan), 2717 g (SD450 g) in 2013 and 2713 g (SD 452 g) in 2018. The Central American site (Guatemala) had a mean BW intermediate between the African and south Asian sites, 2928 g (SD 452) in 2013, and 2874 g (SD 448) in 2018. The low birth weight (LBW) incidence was highest in the south Asian sites (India and Pakistan) and lowest in the African sites (Kenya and Zambia). The size of regional differences varied somewhat over time with slight decreases in the gap in birth weights between the African and Asian sites and slight increases in the gap between the African and Central American sites.

**Conclusions:**

Overall, BWmeans by global region did not change significantly over the 5-year study period. From 2013 to 2018, infants enrolled at the African sites demonstrated the highest BW means overall across the entire study period, particularly as compared to Asian sites. The incidence of LBW was highest in the Asian sites (India and Pakistan) compared to the African and Central American sites.

*Trial registration* The study is registered at clinicaltrials.gov. ClinicalTrial.gov Trial Registration: NCT01073475.

## Background

The weight of an infant at birth (BW) is a crucial anthropometric measurement associated with infant mortality [2–4]. Population BW statisticsare important measures of overall population health. However, in low- and low-middleincome countries (LMICs), BWs are not always measured, and when measured, they are often obtained and recorded inaccurately. Ideally, BW is measured within the first hours after delivery, before significant postnatal weight loss has occurred [1].

A newborn is defined as having normal BW if weight at birth is ≥ 2500 g. Low birth weight (LBW), as defined by the World Health Organization (WHO),isa weight at birth that is less than 2500 g (up to and including 2499 g). Infants with BW < 2500 g are further categorized into low birth weight (LBW), 1500–2499 g; very low birth weight (VLBW), 1000–1499 g; and extremely low birth weight (ELBW) < 1000 g [1]. There is an inverse relationship betweenBW and mortality; newborns with LBW have a higher risk of neonatal mortality and are also at risk for stunting, poor neurodevelopment, and adult-onset diseases [2–4]. Worldwide, an estimated 15–20% of all newbornsweigh < 2500 g at birth [5]. This translates to more than 20million births a year. TheWHO has a goal to reduce the LBW rate by 30% by the year 2025 [6]. In certain regions, there has been an increase in the incidence of LBW deliveries [7]. LMICs carry the highest burden of LBW infants. In 2015, three-quarters of the world’sLBWnewborns were born in three regions: south Asia (47%), eastern and southern Africa (13%) and west and central Africa (12%) [5].

In the recent past, data from high-income countries such as the United States and the United Kingdom recorded an increasing trend in mean BW, with a concurrent decrease in the prevalence of LBW [8, 9]. This finding prompts the question as to whether a similar trend is occurring in LMICs.Exploring temporal trends in BW are important to health care policymakers, especially if there are changes in or regression in medical care or nursing practices, or patterns related to health service access [10]. For example, lack of, or late access to comprehensive antenatal care, which is common in LMICs [11], is correlated with a higher risk of pregnancy and newborn complications, including LBW. Improving rates of prenatal care is associated with decreases in the risk of premature birth and LBW [12].

A major challenge in monitoring the incidence of LBW is that about 60% of newborn babies in LMICs are not weighed nor have BWre corded [5]. Population-based survey data often rely on retrospective maternal recall and modeled estimates, with statistical methods to adjust for underreporting and misreporting of BW. By contrast, the Global Network prospectively collectsBW data in a population-based maternal and newborn health registry (MNHR) insix sites within five LMIC’s from sub-Saharan Africa (Kenya and Zambia), south Asia (Belgavi and Nagpur India; Pakistan), and Central America (Guatemala) [13]. The purpose of this study was to examine trends and regional variation of documented BW and LBW categories over time and to explore possible factors related to those trends in the Global Network MNHR.

## Methods

**Fig. 1:**
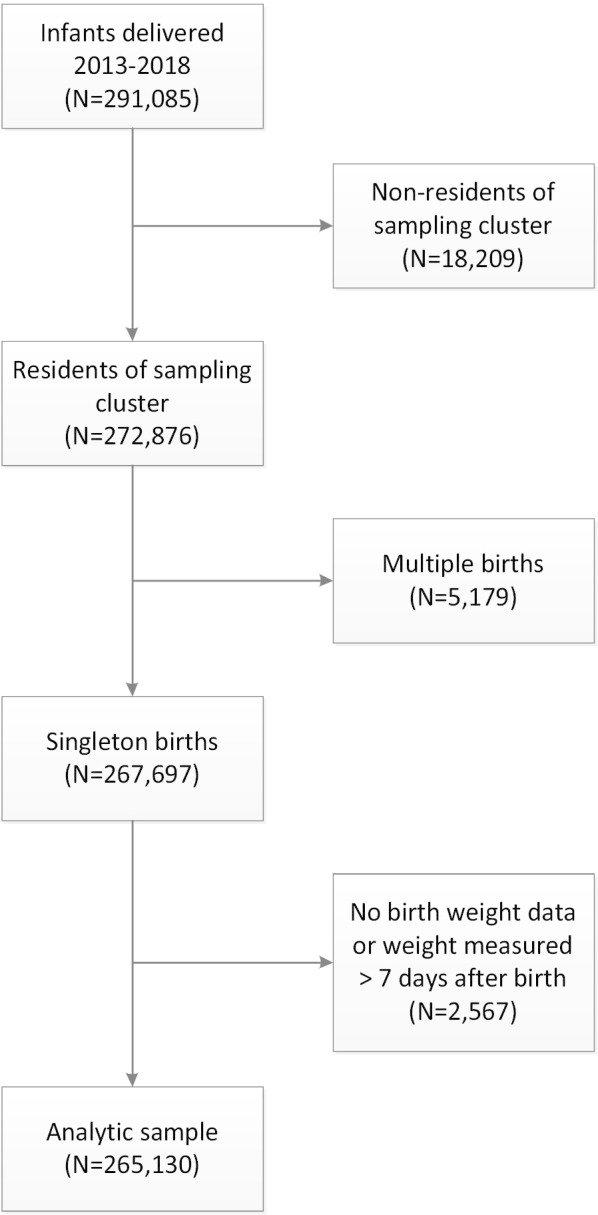
Sample selection

We performed a longitudinal cohort analysis of all infants born to mothers enrolled in the MNHR of the Global Network between 2013 and 2018. For the analysis, all deliveries with a measured BW, obtained between day 0 and day 7 were included. We excluded multiple births, miscarriages, medically terminated pregnancies, and pregnancies of women living outside the predefined study cluster (Fig. 1). We also excluded from our analyses clusters within sites that started after 2013, or were closed prior to 2018.

For infants born in a health facility, the weight recorded by facility personnel was abstracted from the medical record. For infants born at home, study personnel visited the home and obtained the weight. These weights were measured by the study personnel, or in the case of Kenya by the village elder trained for the task using standard scales [14]. It is however important to note that, in most sites, accurate gestational estimation was not possible, and therefore not included in the analysis.Thus, it is not possible to say whether the birthweights were appropriate for gestational age or not.

### Data analysis

We summarized maternal and neonatal demographic characteristics by year of enrollment. To examine possible demographic changes over time, we compared the characteristics of neonates born in 2013 to those in 2018, using t tests for continuous variables and chi-square tests for categorical variables.

Means and standard deviations (SDs) for BW were computed by region and year. In addition, to account for possible demographic differences across the regions, we computed adjusted mean BWs by region and year, controlling for the following demographic characteristics: maternal age, education, parity, weight, height, infant sex, and time between birth and weight measurement. To compute the adjusted means, we fit a linear mixed-effect regression model of BWby region, year, and region by year interaction, controlling for demographic characteristics and including sampling cluster as a random effect. In addition, we tested for interactions between year and demographic characteristics to determine if birth weights changed for different demographic subgroups over time. In Kenya, maternal height was not routinely measured between 2013 and 2017, hence for this and other missing values on control variables (i.e., demographic characteristics), multiple imputation techniques was utilized. Analysis performed with and without imputations were similar. Given the large sample sizes, we had a high level of statistical power, and therefore, even very small effects were found to be statistically significant. To determine whether significant changes in mean birth weights from 2013 to 2018 were meaningful, we examined Cohen’s d as a measure of effect size for which values of 0.2–0.4 are considered small effects, 0.5–0.7 are medium effects, and 0.8 or higher are large effects.Allanalyses were conducted using SAS version 9.4.

### Ethical consideration

This study was reviewed and approved by all participating sites’ ethics review committees/boards including review boards at each U.S. partner university and the data coordinating center (RTI International). All women provided informed consent for participation in the study, including data collection and the follow-up visits.

## Results

Between 2013 and 2018, we enrolled 355,625 pregnant women in the MNHR. Of these, 1% (N = 3254) were lost to follow up. Of the 291,085 deliveries captured in the MNHR within the study period, 265,130 (91%) met inclusion criteria (Fig. 1). Of the singleton deliveries (267,697), only 1% (2567) did not have a recorded birthweight in the MNHR.

### Maternal demographic by region

As shown in Table 1, maternal age was generally similar across regions, with Central American women in our sample beingslightly older than African or Asian women.African women had slightly higher percentages of women with primary or secondary schooling. African women were heavier, especially compared to Asian women, and taller, especially as compared to Central American women.

**Table 1:** Demographic characteristics by region

Characteristic	Africa (N = 85,551)	Asia (N = 122,349)	Central America (N = 57,230)	Africa vs. Asia		Africa vs. CA		Asia vs. CA	
				Mean/% diff	p value	Mean/% diff	p value	Mean/% diff	p value
Maternal age (years), Mean (SD)	24.43 (6.09)	24.81 (4.24)	26.01 (6.56)	− 0.38	< 0.001	− 1.58	< 0.001	− 1.20	< 0.001
Maternal age (years), N (%)									
11–19	20,101 (24)	7165 (6)	9772 (17)	18	< 0.001	7	< 0.001	− 11	< 0.001
20–35	60,198 (70)	112,716 (92)	41,572 (73)	− 22	< 0.001	− 3	< 0.001	19	< 0.001
36+	5149 (6)	2454 (2)	5883 (10)	4	< 0.001	− 4	< 0.001	− 8	< 0.001
Nulliparous, N (%)	26,529 (31)	76,189 (37)	17,039 (30)	− 6	< 0.001	1	< 0.001	7	< 0.001
Education, N (%)									
No formal education	3902 (5)	36,886 (30)	7583 (13)	− 25	< 0.001	− 8	< 0.001	17	< 0.001
Primary/secondary	77,313 (90)	73,006 (60)	46,323 (81)	30	< 0.001	9	< 0.001	− 21	< 0.001
University	4230 (5)	12,394 (10)	3323 (6)	− 5	< 0.001	− 1	< 0.001	4	< 0.001
Maternal weight(kgs), Mean (SD)	60.19 (9.51)	46.68 (8.05)	56.70 (9.48)	13.51	< 0.001	3.49	< 0.001	− 10.02	< 0.001
Maternal height(cm), Mean (SD)	158.74 (6.72)	152.93 (5.65)	147.10 (5.45)	5.81	< 0.001	11.64	< 0.001	5.83	< 0.001
Male infant, N (%)	43,179 (50)	63,443 (52)	29,175 (51)	− 2	< 0.001	− 1	0.052	1	< 0.001
Facility birth, N (%)	65,281 (76)	108,573 (89)	33,135 (58)	− 13	< 0.001	− 18	< 0.001	31	< 0.001
Number of days between birth and birth weight measurement, Mean (SD)	0.32 (1.04)	0.51 (0.97)	1.07 (1.89)	− 0.19	< 0.001	− 0.75	< 0.001	− 0.56	< 0.001

*CA* Central America

### Birth weight difference by time period and region

Eighty five percent of infants in the sample were weighed withintwo days after birth. Mean BW by region and year are shown in Table 2. Mean changes in BW (grams) from 2013 to 2018 by region were: Africa (36.51, SD = 456.00); Asia (3.86, SD = 451.30); and Central America (53.07, SD = 450.20). Change in birth weight over time was not statistically significant for Asia (p = 0.389). While the changes in mean BW from 2013 to 2018 were statistically significant for Africa and Central America (p < 0.001), these changes did not reach the threshold for even a small effect based on Cohen’s d, suggesting that BW generally remained stable over time: Africa (d = 0.08), Asia (d = 0.01), and Central America (d = 0.12).

**Table 2:** Birth weights (g) by region and year

Year	Africa	Asia	Central America (CA)	Africa vs. Asia		Africa vs. CA		Asia vs. CA	
Unadjusted	Mean (SD)	Mean (SD)	Mean (SD)	Mean diff. (SD)	p value	Mean diff. (SD)	p value	Mean diff. (SD)	p value
2013	3186 (463)	2717 (450)	2928 (452)	469 (455)	< 0.001	258 (459)	< 0.001	− 211 (451)	< 0.001
2014	3172 (454)	2730 (472)	2922 (451)	441 (465)	< 0.001	249 (453)	< 0.001	− 192 (465)	< 0.001
2015	3168 (458)	2717 (482)	2909 (478)	451 (473)	< 0.001	258 (466)	< 0.001	− 193 (481)	< 0.001
2016	3148 (465)	2716 (477)	2897 (440)	432 (472)	< 0.001	251 (455)	< 0.001	− 181 (465)	< 0.001
2017	3149 (467)	2714 (464)	2889 (456)	434 (465)	< 0.001	260 (462)	< 0.001	− 174 (461)	< 0.001
2018	3149 (449)	2713 (452)	2874 (448)	436 (451)	< 0.001	275 (448)	< 0.001	− 161 (451)	< 0.001

Year	Africa	Asia	Central America (CA)	Africa vs. Asia		Africa vs. CA		Asia vs. CA	
Adjusted	Mean (SE)	Mean (SE)	Mean (SE)	Mean diff. (SE)	p value	Mean diff. (SE)	p value	Mean diff. (SE)	p value
2013	3105 (13)	2757 (10)	2918 (18)	348 (17)	< 0.001	187 (22)	< 0.001	− 161 (20)	< 0.001
2014	3100 (13)	2755 (10)	2909 (17)	345 (17)	< 0.001	191 (22)	< 0.001	− 154 (20)	< 0.001
2015	3095 (13)	2753 (10)	2901 (17)	342 (17)	< 0.001	194 (22)	< 0.001	− 148 (20)	< 0.001
2016	3089 (13)	2751 (10)	2892 (17)	339 (17)	< 0.001	197 (22)	< 0.001	− 141 (20)	< 0.001
2017	3084 (13)	2748 (10)	2883 (17)	335 (17)	< 0.001	201 (22)	< 0.001	− 135 (20)	< 0.001
2018	3078 (13)	2746 (10)	2874 (18)	332 (17)	< 0.001	204 (22)	< 0.001	− 128 (20)	< 0.001

Adjusted means obtained from models with the following variables: year, region, year × region interaction, maternal age, parity, year × parity interaction, education, maternal weight, maternal height, infant sex, and time between birth and weight measurement

**Fig. 2:**
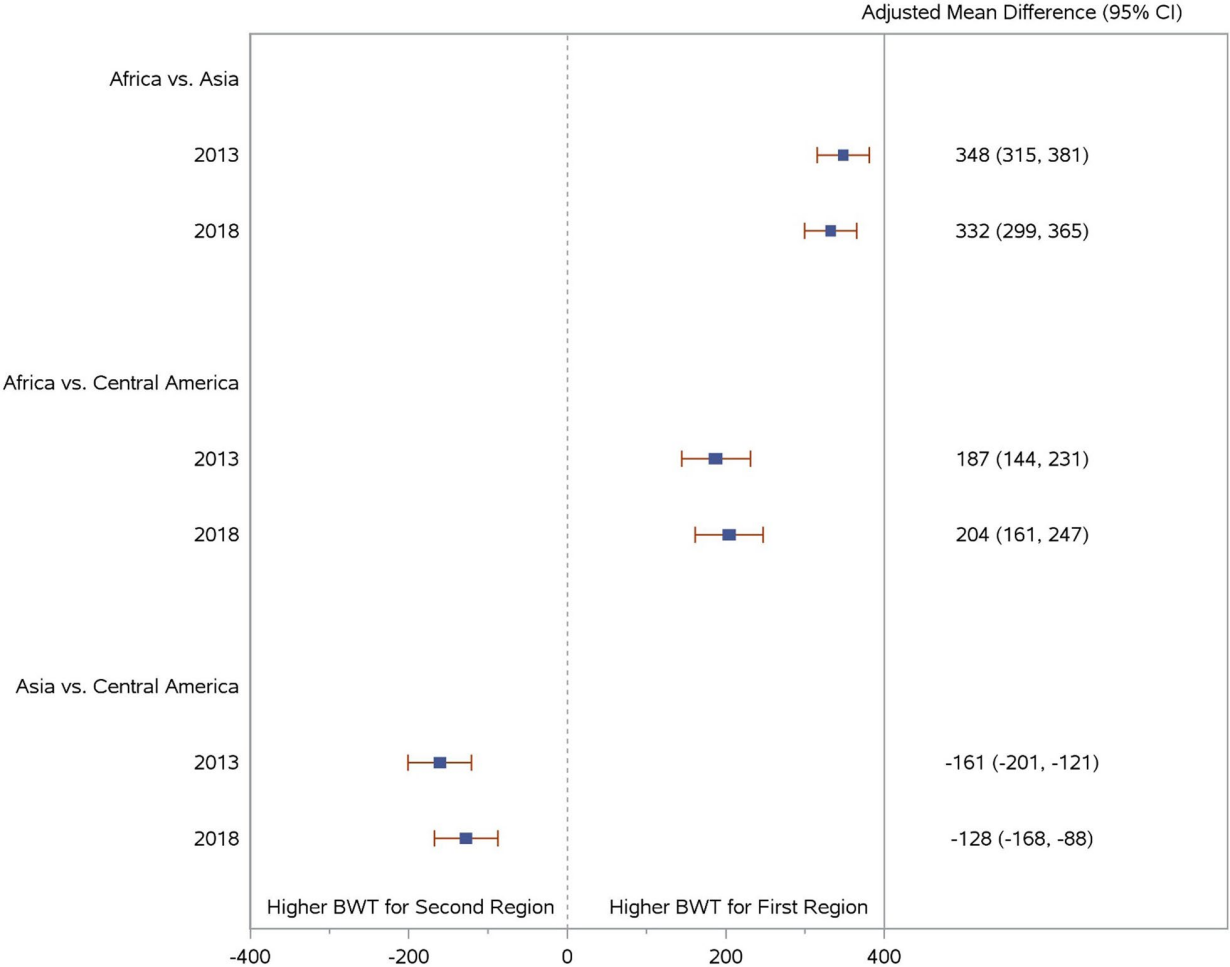
Regional differences in birth weight (g) in 2013 and 2018. Adjusted means obtained from models with the following variables: year, region, year × region interaction, maternal age, parity, year × parity interaction, education, maternal weight, maternal height, infant sex, and time between birth and weight measurement

Birth weights of African newborns were consistently greater than that of Central American infants, which were likewise greater than BWs of Asian neonates. This pattern remained when BW was adjusted for region, year, and maternal demographics, although the size of the mean differences between regions changed slightly over time (Fig. 2).

### Birth weight categories by region

**Fig. 3:**
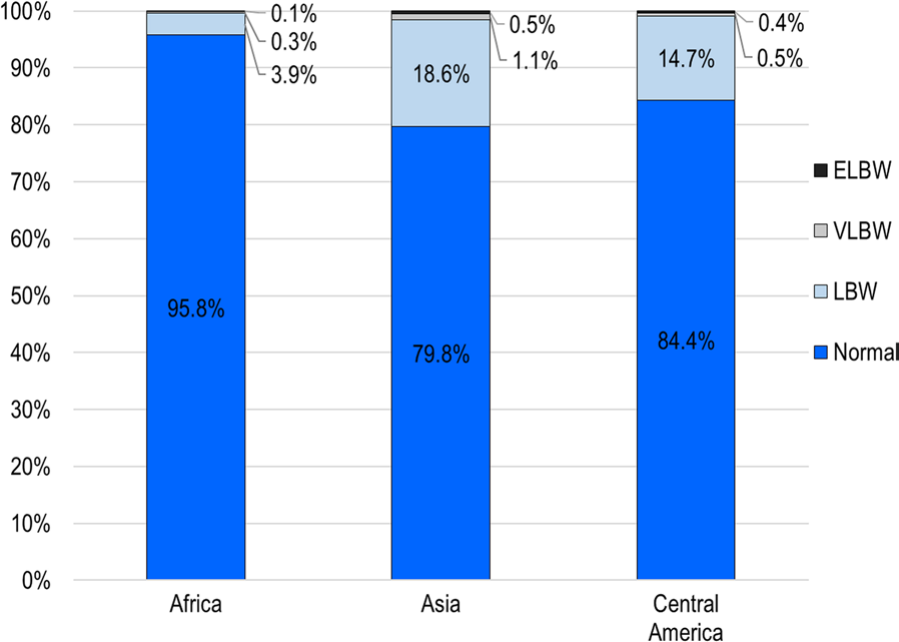
Distribution of birth weight categories by region

Consistent with the pattern seen for mean BW,the African sites had the highest percentage of normal BW (95.8%), hence the lowest percentage of all low BW categories (3.9% LBW, 0.3% VLBW, and 0.1% ELBW; Fig. 3). The Central American site was intermediate, with 84.4% normal BW and 15.6% across all LBW categories, and the Asian regional site had the lowest percentage of normal BW (79.8%) and highest percentages of births in all LBW categories (20.2%; Fig. 3).

## Discussion

We examined trends, and regional differences in mean BW, between 2013 and 2018, of all newborns from six sites in five LMICs enrolled inthe Global Network MNHR. Overall, after controlling for maternal demographic characteristics, there appeared to be a consistent pattern of regional differences across the time period. The mean BW was generally found to be highest in the African regional site (Zambia and Kenya) as compared to the other regional sites, of South Asia (India and Pakistan) and Central America (Guatemala). Across the study period, there were slight changes observed in the size of these disparities over time, with the gap between the African and Asian sites decreasing, and the gap between the African and Central American sites increasing. These observations, however, may not be generalizable to the regions on whole, since the presence of the registry in these clusters may have exerted an influence (Hawthorne effect) on pregnancy outcomes over time.

The highest annual LBW rates were recorded in the Asian sites at 20.2% (18.6%, 1.1%, and 0.5% for LBW, VLBW and ELBW respectively) and the Central American site at 15.6% (14.7%, 0.5%, 0.4% for LBW, VLBW and ELBW respectively). This is consistent with a 2019 UNICEF report, in which the LBW rate in south Asia was 28%. However, the prevalence of LBW in Latin American was report to be 8.7%, which was almost half of what our study reports [5]. Similar findings have also been reported in the WHO multicenter Growth Reference Study [15]. The LBW rate in the African sites in our study was 4%. This result is similar to the proportion (3.5%) reported in the Intergrowth21st study [16], but differs from 13% reported in the 2019 UNICEF report [5]. A possible explanation for this difference is that the data used for the UNICEF report were obtained from multiple sources and subjected to modeling. Up to 28% of the births in the UNICEF study hadno weight recorded, with the highest rates of missing BW data werereported to have occurred in Africa, where the rate ofmissing birthweight data was estimatedto be over 50% [5]. By contrast, in the Global Network’s prospective, population-based MNHR from 2013 to 2018, 85% of the newborns were weighed at or within 2 days of birth.

Usually,the causes of LBW deliveries are multifactorial. Genetic and environmental factors play a significant role. Parity, low socioeconomic status, marital status, maternal age, nutritional status, maternal body mass index (BMI), maternal health status, smoking, alcohol intake, and prevailing infections such as from malariahave all been associated with BW outcomes [16–19]. There exist regional differences in the prevalence of certain diseases, such asmalaria, which has been reported to increase the odds of LBW deliveries [20–22]. Maternal genes in addition to other factors determine the intrauterine environment andmay vary with region and race [16]. In a study examining birth outcomes ofFilipina mothers living in Canada, BW among their babies was lower compared to infants of native Canadian mothers in the same environment [23]. Maternal diseases (e.g., diabetes and hypertensive disease) can also affect weight of a newborn. Socio-economic status and other associated factors have been reported to influence BW.These determinants of BW vary across ethnic populations. It is still unclear to what extent the lower BW of some ethnic minority populations can be explained by these determinants [24, 25].

Some studies report a direct relationship between maternal age and BW. This relationship was demonstrated in a large cohort study in the United Statesbetween 2005 and 2014 [17]. The majority of mothers in our cohort were aged 20–35 years, with African sites and Asia sites having a lower maternal age compared to the Central American site. However, our Asian sites had the lowest rate of teenage pregnancies compared to the other two regions.

One limitation of our study is that not all BWs were measured on the same day, immediately after birth. The time a newborn weight is obtained may affect the recorded BW. However, in our study, this limitation is attenuated.The vast majority of all babies included in the analysis were weighed within 48 h of birth, and, for the entire sample, birthweights were acquired within one week of delivery. However, regional differences in time of weighing were also observed; African sites weighed the newborns closer to time of birth as compared to the Asian and Central American regional sites.

An additional potential source of bias in the results is the population of women who were entered into the study, but were lost to follow-up before the birth of the infant and measurement of BW. Our rates for loss-to-follow-up were quite low (1%); however, it is possible that preterm and LBW infants are over-represented among infants lost to follow-up, resulting in bias towards larger infants in the measured and reported population. Also, stillbirth and early neonatal deaths were likely to have their birthweights estimated instead of measured.

Observer errors have been reported in some studies of BW, as a result of digit preference. As an example, weights ending in 5 (five) or 0 (zero) tend to be preferred, as well as weights of multiples of 100. This is especially problematic when a continuous BW variable is categorized. For instance, an infant with a measured BW of 2492 g may be recorded as 2500, and hence categorized as a normal BW rather than LBW. Digit preference and rounding errors may result in over or underestimation, and therefore may affect observed BW trends [26]. Some infant weighing scales also tend to have readings to the nearest 50 g or nearest 100 g, and this may underestimate the LBW rates. In our cohort, the process of obtaining and documenting birth weight is subject to this potential error.

A final limitation of our study is that the data were prospectively obtained from relatively small, discrete geographical areas (clusters) within each country. Hence, the data may not be representative of the country or region as a whole. However, as compared to other methods and data sources (e.g., Demographic Health Surveys) we enrolled an extremely large number of participants, prospectively, and followed standard procedures in obtaining and documenting weight, across sites, throughout the study period.

## Conclusions

In a prospective, population-based, longitudinal cohort study of birthweight among three global regions, the observed BW meanshad no significant changeover time in aggregate or by region. In addition, theBWmeans recorded for African sites, as compared to the Asian sites, remained consistently higher.

The LBWrate was consistently higher in the Asian sites as compared to the African sites. The incidence of LBW observed for the two African sites in the MNHR was lower than that reported for other global estimates.

As compared to past regional estimates of BW, those obtained in the current study were determined from a very large sample of actual birthweights measured within sevendays of delivery. BW is impacted by a variety of complex maternal and environmental characteristics; future investigations should focus on determining the mechanistic underpinnings of regional and site differences in BW observed in this study.

## Data Availability

The dataset generated and analyzed during this study is not yet publicly available due to ongoing data analysis but it will be available in the NHCHDdata and specimen Hub. Request for data prior to public release will be handled by the author.

## Abbreviations

BW: Birth weight
ELBW: Extremely low birth weight
GN: Global Network for Women's and Children's Health Research
LBW: Low birth weight
LMIC: Low and middle-income country
MNHR: Maternal and newborn health registry
SD: Standard deviation
VLBW: Very low birth weight
WHO: World Health Organization
